# Evaluating Agricultural Sustainability and Green GDP in China: An Emergy Analysis

**DOI:** 10.3390/ijerph192416735

**Published:** 2022-12-13

**Authors:** Jiangfeng Hu, Jingjing Lyu, Xinyuan Zhang

**Affiliations:** 1 Chongqing Academy of Social Sciences, Chongqing 400020, China; 2 School of Business, Northeast Normal University, Changchun 130117, China; 3 School of Taxation, Jilin University of Finance and Economics, Changchun 130117, China

**Keywords:** China, agricultural sustainability, environmental cost, green GDP, emergy analysis

## Abstract

Agricultural sustainability is the foundation and a guarantee of sustainable human reproduction. The scientific assessment of China’s agricultural sustainability is a prerequisite for properly resolving the conflict between short-term economic interests and long-term ecological security. This paper uses the emergy analysis method to estimate agricultural sustainability in China and further calculates the agricultural environmental cost and green GDP. The results show that China’s agricultural emergy yield rate (EYR) is generally greater than 1. This means that more emergy is obtained in relation to renewable and non-renewable inputs from human activity, which also indicates that China’s agricultural agroecosystem is characteristic of a profound transition from a self-supporting tradition to a modern industry based on external economic resource consumption. In contrast, China’s agricultural growth is mainly driven by the input of a large amount of non-renewable resources, which makes the environmental loading rate (ELR) increase year by year, resulting in the deterioration of China’s agricultural emergy sustainability index (ESI). China’s agricultural green GDP accounts for about 94.4% of traditional GDP, which means that the average agricultural environmental cost is about 5.6%, mainly from land loss, accounting for 48.23% of the environmental cost.

## 1. Introduction

Since the reform and opening up, the Chinese government has made the increase in grain output the main goal of agricultural economic development. Grain production has increased from 305 million tons in 1978 to 618 million tons in 2017, solving the problem of food and clothing for about one-fifth of the world’s population. However, China’s agriculture unilaterally pursues production growth, and has not paid attention to the problem of nonpoint source pollution caused by the low-efficiency and large-scale use of chemical agricultural materials. According to data, the utilization rate of chemical fertilizers and pesticides in China is less than 1/3, the recycling rate of plastic film is less than 2/3, the effective treatment rate of livestock and poultry manure is less than 50%, and the burning of straw and the eutrophication of water bodies are serious [1,2]. It is estimated that China’s annual economic loss due to water pollution is about 150 billion yuan [3,4]. Moreover, in order to feed the continuously growing population in the future, China’s agricultural ecological environment will face greater bearing pressure. Therefore, a scientific assessment of China’s agricultural sustainability is not only a prerequisite for properly resolving the conflict between short-term economic interests and long-term ecological security, but also helps provide a reference for the Chinese government to formulate agricultural sustainable development policies.

At present, some scholars mainly use Data Envelopment Analysis (DEA), life cycle analysis, and emergy analysis to evaluate the ability of agricultural sustainable development. Han et al. [5] and Han et al. [6] used the pollutants total phosphorus (TP), total nitrogen (TN), and chemical oxygen demand (COD) produced during agricultural production as unintended outputs and found that environmental pollution had a large efficiency loss on agricultural development and failed to meet the requirements of “good and fast” development of the national economy. Xu et al. [7] and He et al. [8] used agricultural carbon emissions as an unexpected output to measure agricultural carbon emissions performance. Although the inclusion of unintended outputs within the DEA framework allows accounting for green growth performance in agriculture, it is still relatively arbitrary with regard to issues such as pollutant selection and quantification [9]. Moreover, it also does not satisfy the law of conservation of matter [10,11,12,13], thus making it highly susceptible to biased results. In terms of sustainability evaluation, the Life Cycle Sustainability Assessment (LCSA), a comprehensive evaluation method including Life Cycle Assessment (LCA), Life Cycle Cost (LCC), and Social Life Cycle Assessment (SLCA), has been widely used in agricultural sustainability studies. Examples include studies involving composite landscape indicators on Swiss farms [14], sustainability assessment of Italian olive groves [15], and SLCA of Swiss farms [16]. However, LCA lacks consideration of ecosystem goods and services, thus neglecting the contribution of natural resources to agricultural activities.

In order to comprehensively evaluate the ecological economic system, Odum [17] established the theory of emergy analysis and introduced this method into the field of ecological environment accounting. The emergy analysis method starts from the perspective of regional biosphere energy movement, expresses all energy consumed by a certain resource or product in the process of formation or production by emergy, and establishes a systematic sustainable performance value evaluation system on this basis. Compared with other environmental accounting methods, the advantages of the emergy analysis method lie in the following three points. Firstly, it enables the transformation of all the different categories of energy, resources, products, and even labor and services, which are incomparable and difficult to account for, into a uniform scale of “solar emergy emjoules (sej)” [17,18]. It facilitates the integrated assessment of short-term economic benefits and long-term sustainable development performance. Secondly, the emergy theory is based on the laws of material and energy flow in the biosphere and is more convincing in reflecting the true value of the agroecological environment. Thirdly, emergy analysis is not only an important method of environmental accounting, it also provides a detailed portrayal of the material flows and energy transfers in the agricultural production process, making it an important tool for system analysis and evaluation. Due to the many advantages of emergy analysis, more and more studies are beginning to use this method to assess the sustainability level of agricultural production [19,20], environmental damage from agricultural non-point source pollution [21], and the sustainability of cultivated land in China [22].

This paper contributes to the literature in two major ways. Firstly, this paper uses emergy analysis to evaluate the ecological and environmental cost of agricultural nonpoint source pollution. Excessive use of chemical fertilizers not only cannot be effectively absorbed by crops, but also drains into water bodies and causes non-point source pollution. Agricultural non-point source pollution, also known as diffused pollution, mainly refers to soil fertilizer loss during agricultural production, livestock breeding discharge, solid waste discharge, aquaculture discharge, etc. In addition, for a long time, China’s agricultural straw has been disposed of by stacking and burning, which may also induce agricultural nonpoint source pollution. However, existing studies have not included pollutants in the emergy analysis when evaluating agricultural sustainability, which may overestimate the agricultural green development performance. To fill this gap, this paper refers to Zou et al. [2], Han et al. [6], and Qu et al. [23] using the unit survey method to calculate the pollutant emissions in the agricultural production process, and then uses the emergy analysis method to calculate the environmental cost of agricultural nonpoint source pollution.

Secondly, this paper calculates agricultural green GDP. Existing research mainly uses the emergy method to assess economic sustainability [21,22,24,25], and has not yet fully utilized the advantages of the emergy analysis method, namely, it can compare all different types of energy, resources, products, and even labor and services, which are incomparable and difficult to account for, into a uniform scale of “solar emergy emjoules (sej)”. In this way, the emergy can be further linked with the traditional GDP, and the agricultural green GDP can be calculated. In recent years, a small number of scholars have also assessed green GDP using emergy analysis [9,26], but not for agriculture and without accounting for the environmental cost. Therefore, this paper further adopts the emergy analysis method to evaluate the agricultural green GDP on the basis of accounting for the environmental cost.

The remainder of this paper is organized as follows. Section 2 describes the methods, variables, and data. Section 3 presents the results and discussion, and Section 4 ends with conclusions and policy implications.

## 2. Materials and Methods

### 2.1. Emergy Analysis

Emergy transformity is used to convert various ecological flows in the natural, economic, and social subsystems into a unified emergy dimension, and then the macroeconomic value is measured by the converted emergy currency value (that is, the market currency value corresponding to the emergy). According to the emergy analysis steps, cities in China are taken as the research areas. (There are four levels of administrative divisions in China: province (autonomous region, municipality directly under the central government), prefecture-level city (city), country, and township (town-village)). Due to sample size, we focus our analysis on the prefecture-level cities that have a relatively large number of observations for each city.

**Figure 1:**
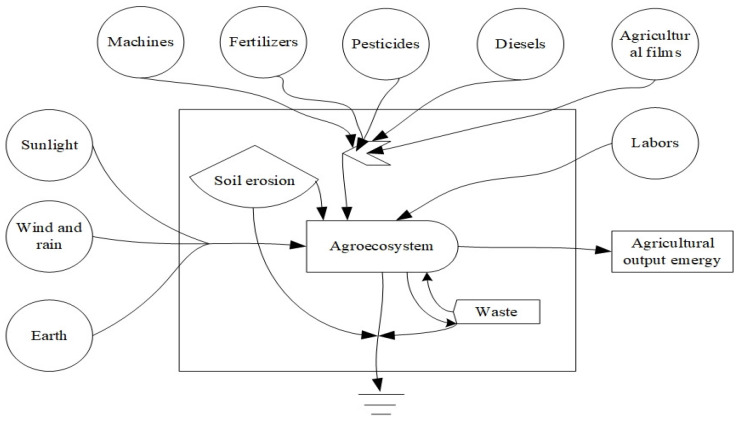
Emergy system diagram of the agricultural system.

Firstly, the spatiotemporal boundary of the agroecosystem is identified and graphical language is used to depict the input–output system. As shown in Figure 1, the larger matrix box represents the boundary and extent of the entire ecosystem. The left side of the diagram represents natural renewable inputs, including sunlight, wind, rain, and Earth rotation; the upper part is human economic and social inputs, including machines, fertilizers, pesticides, diesel fuel, agricultural film, and labor; the right side is agricultural outputs, including intended and unintended outputs (pollution); and finally, the soil erosion within the box is the system’s own output.

Secondly, the underlying variables are categorized. According to the characteristics of the agroecological economic system, it can be divided into six categories: Renewable natural resources, non-renewable natural resources, renewable resource products, externally imported non-renewable resources, externally imported renewable resources, and waste streams (Table 1). Since sunlight, wind, and rain are directly or indirectly derived from solar energy, in order to prevent double counting, according to the suggestion of Odum [17], this paper only uses the project with the largest emergy value as the renewable resource emergy input.

Thirdly, a system emergy analysis table is prepared that includes raw data, transformity, and energy consumption of each element. Transformity is not only a “transformity factor” that converts an initial substance, service, or energy into an emergy, but also reflects the quality or level of different energies to a certain extent. The higher the transformity, the higher the quality or level of energy contained in the substance or service. The formula for calculating the solar emergy of various ecological flows is:

(1) $$E_{m}=\sum f_{i}\times {\mathrm{UEV}}_{i}i=1,\cdots ,n\;$$

where $E_{m}$ represents the total solar emergy of the system, $f_{i}$ is the material or energy flow of the i-th input or output, and ${\mathrm{UEV}}_{i}$ is the transformity (as shown in Table 2).

### 2.2. Variables

#### 2.2.1. Agricultural Sustainability

At present, there are many indicators for evaluating agricultural sustainability and no uniform criteria have been developed. The main reason is that most studies have different conceptions of sustainable agricultural development. Some scholars point out that sustainable agriculture describes crop management approaches that address the interdependent goals of increasing or at least maintaining yields while protecting the environment, conserving natural resources, and mitigating climate change. Hoang et al. [11] argue that sustainable agriculture should provide sufficient nutritious food for all while reducing environmental risks and enabling agricultural producers to earn substantial incomes. Some scholars also define agricultural sustainability as an increase in food production that neither hinders resource recycling nor leads to ecological degradation. Xie et al. [22] place more emphasis on the compatibility between agricultural production efficiency and ecological protection.

It is clear from the definition that the sustainability of an agricultural system is a complex and integrated concept that cannot be measured by a single indicator. Comprehensive analysis of multiple emergy indicators can better reveal the quality of sustainable intensification of agroecological economic systems. In view of this, we refer to Baráth and Fertő [32] and Yuan et al. [33] for a comprehensive measure of the intensity of agricultural sustainability in China using three comprehensive indicators: (1) emergy yield rate (EYR), the greater the value, the greater the contribution of external resources to create available resources; (2) environmental loading rate (ELR), a larger ELR indicates that agricultural production uses more non-renewable resources relative to renewable resources; and (3) emergy sustainability index (ESI), the higher the value, the greater the sustainable development capability. The variables are defined in Table 3.

#### 2.2.2. Agricultural Green GDP

This paper refers to the methods of He et al. [9], Wei et al. [26], and uses emergy analysis to estimate China’s agricultural green GDP.

(2) EC=GDPU+YO 

where $E_{C}$ is the monetary value of the unit emergy.

Thus, the agricultural green GDP can be obtained:

(3) $$\mathrm{Green}\;\mathrm{GDP}=\mathrm{Traditional}\;\mathrm{GDP}-E_{C}*\left(N_{0}+Y_{N}\right)\;$$

where green GDP represents the total agricultural production value after deducting the cost of environmental damage.

It should be noted that only when the input becomes pollution due to loss will it cause damage to the environment, and at the same time, this also avoids the repeated deduction of non-renewable resources such as chemical fertilizers. Therefore, this paper does not directly deduct the purchased non-renewable emergy input according to the practice of He et al. [9] and Wei et al. [26]: $\mathrm{Green}\;\mathrm{GDP}=\;\mathrm{Traditional}\;\mathrm{GDP}-E_{C}*\left(N_{0}+F_{N}+Y_{N}\right)$.

### 2.3. Data Source

The data for this paper were obtained from the *China Urban Statistical Yearbook*, the *China County Statistical Yearbook*, the *China Statistical Yearbook*, the *China Rural Statistical Yearbook* and various local statistical yearbooks. This paper uses 306 cities in China from 1996–2017 as the sample. Some of the indicators are described as follows: (1) To eliminate inter-year variation in objects, all variables involving monetary values have been converted to real output value with the base year of 1978. (2) In the energy calculation, the baseline value of emergy is 9.44 × 1024 sej/a; the average annual rainfall is the observed average value of meteorological observation stations in each region; and all relevant data were obtained from the China Meteorological Science Data Sharing Service. The pollutants include chemical oxygen demand (COD), total phosphorus (TP), and total nitrogen (TN) loss, and this paper refers to Zou et al. [2], Han et al. [6], and Qu et al. [23] for the inventory analysis method. (3) The emergy transformity can be seen in Table 2.

## 3. Results and Discussion

### 3.1. Evaluating Agricultural Sustainability Evaluation

#### 3.1.1. Emergy Ecological Footprint

It can be seen from Table 4 that from 1996 to 2017, China’s total agricultural input and total output emergy showed an increasing trend. The input emergy increased from 3.49 × 10^23^ sej in 1996 to 4.10 × 10^23^ sej in 2017, an increase of 17.48%. The total output emergy increased from 5.47 × 10^23^ sej to 7.43 × 10^23^ sej, an increase of 35.83%.

In 1996, renewable resources accounted for 57.82% of the total input emergy, of which 85.73% came from renewable natural resources. In 2017, the proportion of renewable resources in emergy decreased to 49.93%, but the proportion of renewable natural resources in emergy rose to 91.35%, mainly because the input of purchased renewable energy decreased year by year. In contrast to the renewable resource emergy income, the proportion of non-renewable resources emergy input has increased year by year, accounting for 41.95% of the total input emergy in 1996, and reached a peak of 53.02% in 2011. Although the share declined thereafter, it remained high at 50.12% in 2017, indicating that China relies mainly on non-renewable resources to drive its agricultural economic growth. In the non-renewable resource emergy input, the purchased non-renewable emergy input (machinery, fertilizers, pesticides, diesel fuel, and agricultural film) accounted for more than 80%.

From 1996 to 2017, the emergy of agricultural products increased from 5.22 × 10^23^ sej to 7.13 × 10^23^ sej, an increase of 36.59%. Its proportion in total output emergy (YGYO) first dropped from 95.42% in 1996 to 94.66% in 2003, and then rose to 95.89% in 2017. Relatively speaking, the pollutant emergy output increased from 2.5 × 10^22^ sej to 3.06 × 10^22^ sej, an increase of 22.4%, which was lower than the growth rate of the emergy of agricultural products. Its proportion in total output emergy (YNYO) first increased from 4.58% in 1996 to 5.38% in 2003, and then decreased year by year to 4.11% in 2017.

#### 3.1.2. Emergy Yield Rate (EYR)

The Emergy Yield Rate (EYR) represents the contribution of purchased external resources to the agroecosystem, with higher values indicating greater contributions. The minimum EYR value is 1, which means that the system cannot utilize local resources and can only convert the resources of the previous process [34]. In Table 5, the overall EYR of China’s agriculture is 3.2199, which is far greater than 1. This means that the purchased external resources have a positive contribution to China’s agroecological economic system. At the same time, the EYR of this paper is larger than the 1.19–2.35 estimated by Wang et al. [34], 2.08–2.18 estimated by Jiang et al. [28], and 2.16–2.975 estimated by Xie et al. [22]. This may be due to the fact that this paper uses cropping industry data at the level of 306 cities in China, rather than the single region (province, city, and county) or single agricultural product data that the existing research focuses on. In addition, the EYR of the central region is 3.6725, which is higher than that of the western (3.5191) and eastern (2.6118) regions, which means that the output of the agricultural production system in the central region is more efficient and competitive. This is because most of the central provinces are the main grain-producing areas in China (Heilongjiang, Jiangxi, Hunan, Anhui, Inner Mongolia, Hubei, Henan, and Jilin) and undertake the task of ensuring food security. As a result, the central region will increase grain production by investing in modern elements such as machinery, fertilizers, and pesticides.

**Figure 2:**
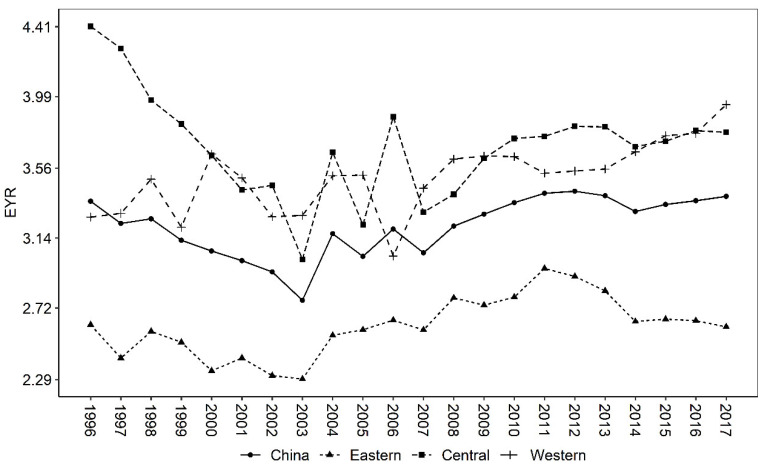
Trends in China’s agricultural EYR, 1996–2017.

The trend in EYR (Figure 2) shows a general trend of decreasing (1996–2003) and then increasing (2003–2017). During 1996–2003, China’s labor outflow accelerated and fertilizer continued to grow, which led to a decline in EYR. In 2003–2017, China’s agricultural trade shifted from surplus to deficit, and food security gradually gained government concern, such as the full abolition of agricultural taxes and encouragement of agricultural investment in 2006. In addition, since 2015, the Chinese government has issued a series of policies related to green development, hoping to reverse the unsustainable situation of agricultural development. It is worth noting that, relative to the central and western regions, the EYR in the eastern region is significantly lower, indicating that the agricultural sustainability in eastern China to convert external resources into usable resources is lower than that of other regions, and even lower than the national level. This is mainly because the main development in the eastern region is industry and commerce, and the proportion of agriculture is relatively small.

#### 3.1.3. Environmental Load Rate (ELR)

The environmental load rate (ELR) is primarily used to measure the pressure on the ecosystem caused by agricultural production. The concept of environmental load suggests that once a resource is used for this environmental service, it cannot be used for other processes [34,35,36]. A larger ELR indicates that agricultural production uses more non-renewable resources relative to renewable resources [18,34,36]. It can be seen from Table 5 that the ELR is relatively small at 0.9035, indicating a greater use of renewable resources in Chinese agriculture. However, in terms of regional comparisons, the ELR is higher in the eastern region (1.0520) than in the central (0.8734) and western (0.7483) regions. This indicates that agriculture in the eastern has greater use of resources and environmental pressures.

**Figure 3:**
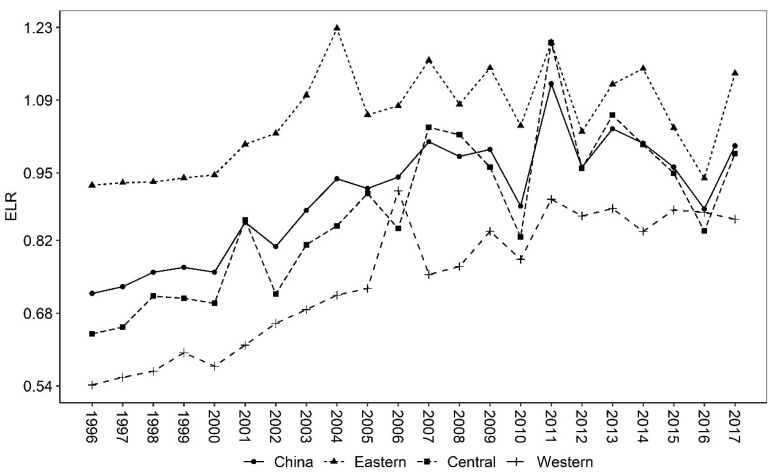
Trends in China’s agricultural ELR, 1996–2017.

The trend in ELR (Figure 3) shows an upward trend, indicating that China’s agricultural growth has been driven mainly by the input of large amounts of non-renewable resources, which has led to an increase in environmental loading pressure year by year. According to relevant studies, during the period 1978–2016, the total amount of fertilizer use in China increased by nearly 6 times [37], accounting for more than 1/3 of global fertilizer use. In addition, agriculture is also the largest water user in China, accounting for 62.6% and 63.1% of the total national water withdrawal in 2001 and 2015, respectively. However, the water use coefficient of irrigated agriculture is only 0.3–0.4, which is much lower than 0.7–0.8 in developed countries [38]. In terms of regional comparisons, the agricultural environmental loading pressure is highest in the eastern region, followed by the central region, and is lowest in the western region. This paper suggests that the possible reasons for this are as follows. On the one hand, most of the western region is utilized for livestock industry due to geological, climatic, and historical factors, while this paper mainly examines the plantation industry and does not include livestock inputs, which may underestimate the inputs of non-renewable resources in the western region, resulting in a low ELR. On the other hand, the relatively low level of economic development in western China does not allow it to afford more modern agricultural inputs such as machinery, fertilizers, and pesticides, which would also make the ELR lower in the western region.

#### 3.1.4. Emergy Sustainability Index (ESI)

The emergy sustainability index (ESI) is mainly used to evaluate the sustainable development capability of the system from the perspective of emergy. The higher the value, the greater the sustainable development capability (Wang et al., 2014). It can be seen from Table 5 that China’s ESI is 3.5637, indicating that China’s overall agricultural sustainable development capability is strong. From the perspective of regional comparison, the ESI in the western region is the largest (4.7027), followed by the central region (4.204), and the smallest is the eastern region at only 2.4868, which is lower than the national level.

**Figure 4:**
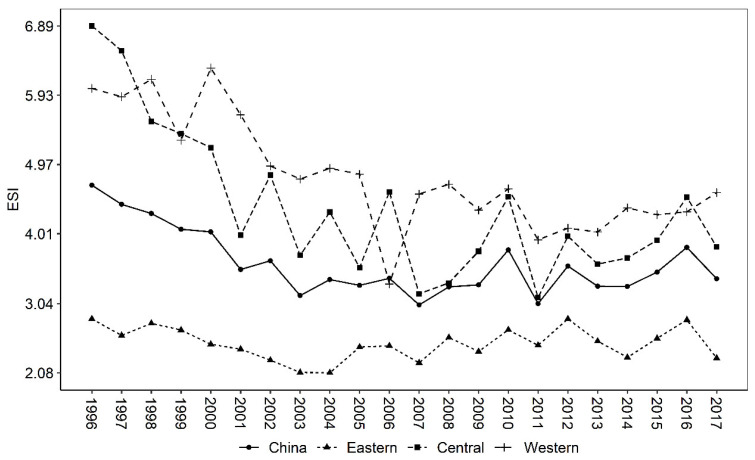
Trends in China’s agricultural ESI, 1996–2017.

The trend in ESI (Figure 4) shows a downward trend, from 4.68 in 1996 to 3.38 in 2017, indicating that China’s agricultural sustainability is decreasing year by year. In terms of regional comparisons, the ESI in the eastern region is the lowest, mainly due to insufficient EYR and high ELR. The central and western regions, on the other hand, have comparable ESI, but for different reasons. The central region is mainly due to a higher EYR than the western region, while the western region is mainly due to a lower ELR than the central region.

#### 3.1.5. EYR, ELR, and ESI

As can be seen from Table 6, the top five provinces with the highest EYR rankings are Heilongjiang (9.109), Xinjiang (9.109), Jiangxi (5.5919), Tibet (5.011), and Hebei (4.7778). The top five provinces with the smallest ELR are Guizhou (0.3317), Jiangxi (0.4268), Hunan (0.4285), and Guangxi (0.4486). The top five provinces with the largest ESI were Heilongjiang (14.1143), Jiangxi (13.1026), Hunan (10.6817), Guizhou (8.3695), and Sichuan (7.0272). Among them, Heilongjiang is mainly due to the highest EYR ranking, while Guizhou is mainly due to the highest ELR ranking. Finally, this paper finds that Hainan has the smallest EYR at 0.8374, indicating that Hainan’s external resource utilization capacity is insufficient. Shanxi has the largest ELR at 3.6669, indicating that Shanxi’s agricultural environment is under greater pressure. Ningxia has the smallest ESI at 0.3556, indicating that Ningxia has the weakest capacity for sustainable agricultural development.

### 3.2. Accounting of Agricultural Environmental Cost and Green GDP

#### 3.2.1. Environmental Cost

Using the aforementioned method, this paper calculated the environmental cost of agricultural production in 306 cities in China from 1996–2017. The results are shown in Table 7. From 1996 to 2017, the average environmental cost of China’s agriculture was about 31.5 billion yuan, accounting for 5.59% of the GDP of traditional agriculture. This shows that ignoring the adverse effects of agricultural production on the ecological environment will overestimate the performance of extensional growth and mislead the formulation of China’s agricultural sustainable development policies. In terms of the composition of environmental loss, the cost of land loss is the largest, as high as 15.21 billion yuan, accounting for 48.23% of the total environmental cost. The TN loss cost is as high as 14.809 billion yuan, accounting for 46.96%. Finally, TP and COD leakage costs are relatively small, at 1.258 billion yuan and 26 billion yuan, respectively. In terms of time trends, the total environmental erosion cost showed an upward and then downward trend. The period of 1996–2014 was the upward phase, from 26.7974 billion yuan to 44.8182 billion yuan; after 2015 was the downward phase, and the total environmental erosion cost was 40.9686 billion yuan by 2017. The possible reason for this is that from 2015 onwards, the Chinese government issued a series of policies on sustainable agricultural development.

#### 3.2.2. Agricultural Green GDP

**Figure 5:**
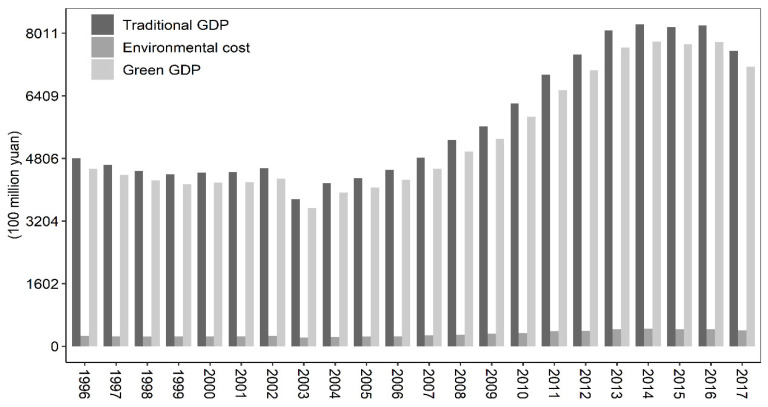
Traditional GDP, environmental cost, and green GDP, 1996–2017.

The trends in traditional agricultural GDP and green agricultural GDP (Figure 5) show an upward trend from 1996 to 2017. The traditional agricultural GDP rose from 480.685 billion yuan in 1996 to 756.28 billion yuan in 2017, an increase of 57.33%. In addition, green agriculture grew faster, from 453.888 billion yuan in 1996 to 715.311 billion yuan in 2017, an increase of 57.6%. This shows that green GDP grows faster than traditional GDP, and the gap between them tends to narrow.

**Figure 6:**
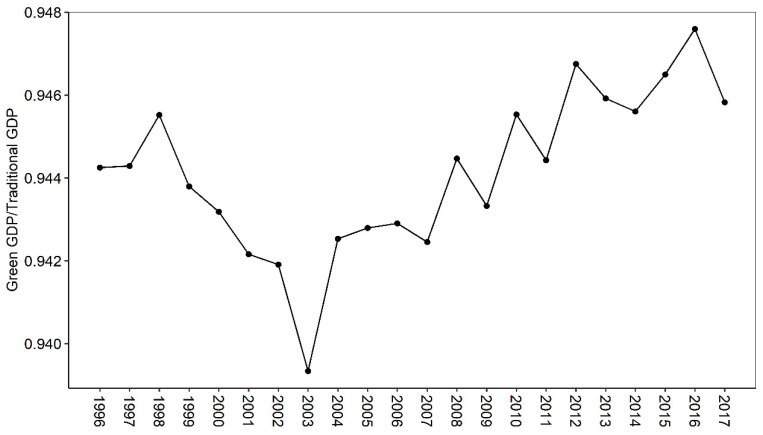
Trends in the rate of China’s agricultural green GDP to traditional GDP, 1996–2017.

From the rate of green GDP to traditional GDP (Figure 6), China’s agricultural growth can be roughly divided into two stages. During the period from 1996 to 2003, the rate of green agricultural GDP/traditional agricultural GDP dropped rapidly, from 0.9443 to 0.9393, indicating that the cost of environmental erosion of agricultural resources in China has increased year by year. After 2003, the rate showed a fluctuating upward trend, rising from 0.9393 to 0.9458, indicating that the cost of environmental loss of agricultural resources in China has been decreasing year by year.

As shown in Table 8, from 1996 to 2017, the five provinces with the highest agricultural green GDP/traditional GDP were Tibet (0.982), Sichuan (0.97), Ningxia (0.965), Guangdong (0.962), and Anhui (0.96). Among them, three provinces are in the western, and one is in the eastern and central regions. In addition, this paper finds that Hebei has the lowest rate of only 0.858, followed by Shandong at 0.893. This shows that, compared with the central and western regions, the ecological and environmental costs of agricultural production in the eastern region are heavier. It seems that the lower the production intensity, the better the environmental rating. This is consistent with the law of diminishing input efficiency. Additionally, Tibet and Ningxia are located in western China, and most of these areas are utilized for livestock due to geological, climatic, and historical factors, while this paper mainly examines the planting industry and does not include pollution from pastoralism, thus possibly overestimating green GDP.

## 4. Conclusions

This paper uses the emergy analysis method to estimate the agricultural sustainability and green GDP of 306 cities in China from 1996 to 2017. The main conclusions can be summarized as follows.

Firstly, China mainly relies on non-renewable resources to drive agricultural economic growth. Although the proportion of pollution energy output has declined, the total amount is still on the rise. Specifically, the proportion of renewable emergy decreased year by year, while the proportion of non-renewable resource emergy input increased year by year. In the non-renewable resource emergy input, the purchased non-renewable emergy input (machinery, fertilizers, pesticides, diesel oil, and agricultural film) is much higher than the land loss emergy. From the comparison of emergy output, the growth rate of emergy value of agricultural products is slightly higher than that of pollution emergy value. The proportion of pollution emergy to total output emergy experienced two stages of first rising (1996–2003) and then decreasing (2003–2017). Secondly, China’s agricultural sustainable development capacity is generally good, but it shows a trend of deteriorating year by year. Specifically, China’s agricultural emergy yield rate (EYR) is generally greater than 1, and decreases in sequence along the central, western, and eastern regions. In terms of changes in EYR, it shows a trend of decreasing (1996–2003) and then increasing (2003–2017). In contrast, because China mainly drives agricultural growth by investing a large amount of non-renewable resources, the environmental load rate (ELR) is increasing year by year, which in turn leads to a yearly decline in China’s agricultural sustainability index (ESI). Thirdly, the gap between China’s agricultural green GDP and traditional GDP shows a U-shaped trend that first widens and then narrows. During the period from 1996 to 2003, the gap between green GDP and traditional GDP increased year by year; from 2003 to 2017, the gap between the two was narrowed year by year. Overall, during 1996–2017, the environmental cost of agriculture in China was about 5.6%, which is very close to the 6% estimated by Tang et al. [3].

Based on the above conclusions, the policy implications of this paper are as follows. Firstly, transforming the crude development of agriculture and increasing green technology innovation and promotion are priorities in agricultural sustainable policy development. The conclusion shows that China mainly relies on non-renewable resources to drive agricultural economic growth, and the inefficient use of non-renewable resources will cause serious agricultural nonpoint source pollution. Therefore, it is necessary to reduce the cost of agricultural resources through advanced technology and factor substitution, such as the promotion of soil testing formula technology, biological pesticide production technology, degradable or reusable film production technology, and shallow burial drip irrigation technology. Secondly, the vigorous development of circular agriculture and promotion of the comprehensive utilization of agricultural waste should be pursued. Agricultural waste is one of the main sources of agricultural nonpoint source pollution. Therefore, research on how to recycle agricultural waste is an inevitable requirement for the sustainable development of China’s agriculture. It is generally believed that planting industry waste and animal manure and residues discharged from aquaculture are major agricultural nonpoint source polluters. Therefore, a combination of planting and aquaculture can be used to balance planting and breeding, turning waste into treasure to realize resource reuse, improve resource utilization efficiency, and reduce pollution emissions. Thirdly, construction of an agricultural green economic indicator accounting system and the consolidation of the “green GDP” assessment mechanism should be priority developments. The cost of China’s agricultural environment is about 5.6% of agricultural GDP, indicating that the traditional accounting system will overestimate China’s agricultural economic development performance. The current “promotion tournament” assessment mechanism for cadres focuses too much on traditional economic development indicators, ignoring the negative impact of sloppy development on the ecological environment. Therefore, the existing local performance appraisal system should be reformed to include environmental weighting in the appraisal system, consolidate leadership responsibilities, and guide local governments to shift from a focus on economic development to a focus on both economic growth and environmental quality improvement, which will truly engage local governments in regional governance.

This paper may have some limitations. Firstly, this paper only focuses on the planting industry, and has not taken livestock breeding into account. Animal husbandry not only generates more greenhouse gases, but it also has a negative impact on the ecological environment if animal waste is not properly treated, which is not conducive to the improvement of agricultural green GDP. Secondly, the Chinese government promises to strive to achieve peak CO_2_ emissions by 2030 and carbon neutrality by 2060. Although industry is the source of greenhouse gases, rapid development of agriculture also plays an important role, and the carbon emissions caused each year should not be underestimated. In the future, we can consider the relationship between resource misallocation and agricultural carbon emissions.

## Figures and Tables

**Table 1:** Agroeconomic ecosystem indicator.

Indicator	Variable	Definition
Renewable natural resources	R	Emergy input such as sunlight, earth rotation, rain, and wind
Non-renewable natural resources	$N_{0}$	Soil erosion emergy
Externally imported non-renewable resources	$F_{N}$	Machines, fertilizers (nitrogen, phosphate, potash, and compound fertilizers), pesticides, diesel fuel, and agricultural film emergy input
Externally imported renewable resources	$F_{R}$	Labor emergy input
Total emergy input	$U\;=\;R\;+{\;N}_{0}+F_{N}+F_{R}$	Gross emergy resources required to support production stems
Renewable resource products emergy output	$Y_{G}$	Emergy output for grain, oilseeds, vegetables, cotton, and fruit
Waste emergy output	$Y_{N}$	Total nitrogen (TN), total phosphorus (TP), and chemical oxygen demand (COD) energy output
Available resource emergy output	$Y_{T}=Y_{G}-Y_{N}$	Renewable resource products emergy output minus waste emergy output
Total emergy output	$Y_{O}=Y_{G}+Y_{N}$	Sum of emergy output of agricultural products and pollutants

**Table 2:** Emergy transformity of China’s agroecosystem.

Indicator	Name	Expression (J/yr)	Transformity	Refs. for Transformity
Renewable natural resources emergy	Solar energy	Sown area (1000 hectares) × Average annual solar radiation intensity (J/m^2^) × Transformity (1.00 sej/J)	1.00 × 10^0^ sej/J	Xie et al. [22], Zhang et al. [27]
	Rain, geopotential	Sown area (1000 hectares) × Average annual rainfall (m) × Water density (1 × 10^6^ g/m^3^) × Average altitude (450 m) × g (9.8 m/s^2^) × Transformity (8.89 × 10^3^ sej/J)	8.89 × 10^3^ sej/J	Xie et al. [22], Zhang et al. [27]
	Rain, chemical	Sown area (1000 hectares) × Average annual rainfall (m) × Evapotranspiration rate (0.57) × Gibbs free energy (4.94 J/g) × Water density (1 × 10^6^ g/m^3^) × Transformity (1.54 × 10^4^ sej/J)	1.54 × 10^4^ sej/J	Xie et al. [22], Zhang et al. [27]
	Earth cycle	Sown area (1000 hectares) × Heat flow per area (1.81 × 10^6^ J/m^2^) × Transformity (2.9 × 10^4^ sej/J)	2.9 × 10^4^ sej/J	Xie et al. [22], Zhang et al. [27]
Non-renewable natural resources emergy	Soil erosion	Sown area (1000 hectares) × Soil loss (1.34 × 10^3^ g/m^2^/yr) × Average organic content (0.56) × Organic content energy (5.4 Kcal/g × 4186 J/Kcal) × Transformity (6.25 × 10^4^ sej/J)	6.25 × 10^4^ sej/J	Zhang et al. [27]
Externally imported non-renewable resources emergy	Machinery	Mechanical power (kW·h) × Mechanical power weight (3.6 × 10^6^ J/kW·h) × Transformity (7.50 × 10^4^ sej/J)	7.50 × 10^4^ sej/J	Zhang et al. [27]
	Nitrogen fertilizer	Nitrogen fertilizer (t) × Energy per unit of nitrogen fertilizer (2.40 × 10^10^ J/t) × Transformity (1.69 × 10^6^ sej/J)	1.69 × 10^6^ sej/J	Jiang et al. [28]
	Phosphate fertilizer	Phosphate fertilizer (t) × Transformity (1.78 × 10^16^ sej/t)	1.78 × 10^16^ sej/t	Jiang et al. [28]
	Potash fertilizer	Potash fertilizer (t) × Energy per unit of potash fertilizer (9.00 × 10^9^ J/t) × Transformity (2.63 × 10^6^ sej/J)	2.63 × 10^6^ sej/J	Jiang et al. [28]
	Compound fertilizer	Compound fertilizer (t) × Transformity (2.80 × 10^15^ sej/t)	2.80 × 10^15^ sej/t	Jiang et al. [28]
Externally imported renewable resources emergy	Labor	Labor number (P) × Average annual working days (150 days) × energy cost per day (506 Kal/8 h) × Transformity (3.80 × 10^5^ sej/J)	3.80 × 10^5^ sej/J	Wang et al. [29]
Renewable resource products emergy output	Grain	Grain (t) × Grain unit energy (1.62 × 10^10^ J/t) × Transformity (8.30 × 10^5^ sej/J)	8.30 × 10^4^ sej/J	Zhang et al. [27]
	Oil-bearing crops	Oil-bearing crops (t) ×Oil-bearing crops unit energy (3.86 × 10^10^ J/t) × Transformity (6.90 × 10^5^ sej/J)	6.90 × 10^5^ sej/J	Zhang et al. [27]
	Vegetable	Vegetable (t) × Vegetable unit energy (2.46 × 10^9^ J/t) × Transformity (2.70 × 10^4^ sej/J)	2.70 × 10^4^ sej/J	Zhang et al. [27]
	Cotton	Cotton (t) × Cotton unit energy (1.88 × 10^10^ J/t) × Transformity (1.90 × 10^6^ sej/J)	1.90 × 10^6^ sej/J	Zhang et al. [27]
	Fruit	Fruit (t) × Fruit unit energy (2.65 × 10^9^ J/t) × Transformity (5.30 × 10^5^ sej/J)	5.30 × 10^5^ sej/J	Zhang et al. [27]
Waste emergy output	TN	TN (t) × Transformity (5.35 × 10^15^ sej/t)	6.66 × 10^5^ sej/J	Pollutants were accounted for using inventory analysis methods Zou et al. [2], Han et al. [6], and Qu et al. [23], while transformity for each pollutant was determined according to Sun et al. [30] and Zhao et al. [31]
	TP	TP (t) × Transformity (8.75 × 10^15^ sej/t)	8.75 × 10^15^ sej/J	
	COD	COD (t) × Calories (3.4 × 10^6^ J/t) × Unit energy (4186 J/kcal) × Transformity (4.84 × 10^6^ sej/J)	4.84 × 10^6^ sej/J	

**Table 3:** Agricultural sustainability variable.

Variable	Expression	Explanation
EYR	$\mathrm{EYR}=Y_{T}/\left(F_{R}+F_{N}\right)$	The ability of external resources to create available resources
ELR	$\mathrm{ELR}=\left(N_{0}+F_{N}\right)/\left(R+F_{R}\right)$	The environmental load intensity of agricultural systems
ESI	ESI=EYR/ELR	The emergy sustainable intensity of agricultural systems

**Table 4:** Results of accounting for agroeconomic ecosystem emergy in China, 1996–2017. (Unit: sej).

Year	R	N_0_	F_R_	F_N_	U	Y_G_	Y_N_	Y_O_
1996	1.73 × 10^23^	2.54 × 10^22^	2.88 × 10^22^	1.21 × 10^23^	3.49 × 10^23^	5.22 × 10^23^	2.50 × 10^22^	5.47 × 10^23^
1997	1.76 × 10^23^	2.59 × 10^22^	2.84 × 10^22^	1.25 × 10^23^	3.55 × 10^23^	5.15 × 10^23^	2.57 × 10^22^	5.40 × 10^23^
1998	1.75 × 10^23^	2.59 × 10^22^	2.91 × 10^22^	1.31 × 10^23^	3.61 × 10^23^	5.40 × 10^23^	2.66 × 10^22^	5.66 × 10^23^
1999	1.79 × 10^23^	2.63 × 10^22^	2.83 × 10^22^	1.35 × 10^23^	3.68 × 10^23^	5.29 × 10^23^	2.68 × 10^22^	5.56 × 10^23^
2000	1.78 × 10^23^	2.61 × 10^22^	2.95 × 10^22^	1.32 × 10^23^	3.66 × 10^23^	5.16 × 10^23^	2.62 × 10^22^	5.42 × 10^23^
2001	1.62 × 10^23^	2.56 × 10^22^	2.72 × 10^22^	1.37 × 10^23^	3.52 × 10^23^	5.17 × 10^23^	2.67 × 10^22^	5.43 × 10^23^
2002	1.80 × 10^23^	2.56 × 10^22^	2.61 × 10^22^	1.41 × 10^23^	3.73 × 10^23^	5.14 × 10^23^	2.72 × 10^22^	5.41 × 10^23^
2003	1.71 × 10^23^	2.52 × 10^22^	2.32 × 10^22^	1.45 × 10^23^	3.64 × 10^23^	4.89 × 10^23^	2.75 × 10^22^	5.16 × 10^23^
2004	1.61 × 10^23^	2.60 × 10^22^	2.30 × 10^22^	1.48 × 10^23^	3.59 × 10^23^	5.65 × 10^23^	2.78 × 10^22^	5.93 × 10^23^
2005	1.79 × 10^23^	2.67 × 10^22^	2.25 × 10^22^	1.60 × 10^23^	3.88 × 10^23^	5.77 × 10^23^	2.92 × 10^22^	6.06 × 10^23^
2006	1.74 × 10^23^	2.72 × 10^22^	2.28 × 10^22^	1.61 × 10^23^	3.85 × 10^23^	6.10 × 10^23^	3.05 × 10^22^	6.41 × 10^23^
2007	1.70 × 10^23^	2.68 × 10^22^	2.19 × 10^22^	1.69 × 10^23^	3.88 × 10^23^	6.07 × 10^23^	3.04 × 10^22^	6.37 × 10^23^
2008	1.80 × 10^23^	2.72 × 10^22^	2.09 × 10^22^	1.71 × 10^23^	3.99 × 10^23^	6.43 × 10^23^	3.06 × 10^22^	6.74 × 10^23^
2009	1.73 × 10^23^	2.83 × 10^22^	2.18 × 10^22^	1.67 × 10^23^	3.90 × 10^23^	6.46 × 10^23^	3.03 × 10^22^	6.77 × 10^23^
2010	2.03 × 10^23^	2.85 × 10^22^	2.18 × 10^22^	1.70 × 10^23^	4.23 × 10^23^	6.69 × 10^23^	3.07 × 10^22^	7.00 × 10^23^
2011	1.58 × 10^23^	2.80 × 10^22^	2.12 × 10^22^	1.74 × 10^23^	3.81 × 10^23^	6.95 × 10^23^	3.12 × 10^22^	7.26 × 10^23^
2012	1.93 × 10^23^	2.85 × 10^22^	2.14 × 10^22^	1.79 × 10^23^	4.21 × 10^23^	7.15 × 10^23^	3.18 × 10^22^	7.47 × 10^23^
2013	1.80 × 10^23^	2.86 × 10^22^	2.11 × 10^22^	1.80 × 10^23^	4.09 × 10^23^	7.15 × 10^23^	3.19 × 10^22^	7.47 × 10^23^
2014	1.90 × 10^23^	2.89 × 10^22^	2.15 × 10^22^	1.84 × 10^23^	4.24 × 10^23^	7.25 × 10^23^	3.21 × 10^22^	7.57 × 10^23^
2015	2.03 × 10^23^	2.90 × 10^22^	1.89 × 10^22^	1.83 × 10^23^	4.33 × 10^23^	7.21 × 10^23^	3.19 × 10^22^	7.53 × 10^23^
2016	2.19 × 10^23^	2.89 × 10^22^	1.89 × 10^22^	1.78 × 10^23^	4.45 × 10^23^	7.08 × 10^23^	3.09 × 10^22^	7.39 × 10^23^
2017	1.87 × 10^23^	2.85 × 10^22^	1.77 × 10^22^	1.77 × 10^23^	4.10 × 10^23^	7.13 × 10^23^	3.06 × 10^22^	7.43 × 10^23^

R: Renewable natural resources, $N_{0}$: non-renewable natural resources, $F_{R}$: externally imported renewable resources, $F_{N}$: externally imported non-renewable resources, U: total emergy input, $Y_{G}$: renewable resource products emergy output, $Y_{N}$: waste emergy output, and $Y_{O}$: total emergy output.

**Table 5:** Regional comparison results on agricultural sustainability in China, 1996–2017.

Variable	China	Eastern	Central	Western
EYR	3.2199	2.6118	3.6725	3.5191
ELR	0.9035	1.0502	0.8734	0.7483
ESI	3.5637	2.4868	4.2047	4.7027

**Table 6:** The provincial comparison results of China’s agricultural sustainability.

Province	EYR	ELR	ESI	EYR Rank	ELR Rank	ESI Rank
**Eastern**						
Zhejiang	3.2223	0.5054	6.3757	16	6	7
Guangxi	2.6935	0.4486	6.0043	18	4	9
Guangdong	2.5242	0.4903	5.1483	19	5	10
Jiangsu	3.8556	0.8541	4.5144	11	14	12
Hebei	4.7778	1.1283	4.2345	5	19	13
Liaoning	4.728	1.2184	3.8804	6	21	15
Fujian	2.2032	0.6093	3.6157	22	9	18
Hainan	0.8374	1.1531	0.7262	27	20	25
Shandong	1.3716	3.1318	0.438	25	25	26
**Central**						
Heilongjiang	9.109	0.6454	14.1143	1	10	1
Jiangxi	5.5919	0.4268	13.1026	3	2	2
Hunan	4.5776	0.4285	10.6817	7	3	3
Anhui	4.1555	0.6709	6.1944	9	11	8
Inner Mongolia	4.3397	1.0603	4.093	8	17	14
Hubei	3.3352	0.8986	3.7115	14	15	16
Henan	3.3744	1.0402	3.2439	13	16	20
Jilin	2.3466	2.6202	0.8956	21	24	23
Shanxi	3.2607	3.6669	0.8892	15	27	24
**Western**						
Guizhou	2.7764	0.3317	8.3695	17	1	4
Sichuan	4.1387	0.5889	7.0272	10	7	5
Tibet	5.011	0.7218	6.942	4	12	6
Gansu	3.5532	0.775	4.5848	12	13	11
Xinjiang	8.4976	2.332	3.6439	2	23	17
Yunnan	2.1387	0.595	3.5943	23	8	19
Shaanxi	2.3994	1.1192	2.1438	20	18	21
Qinghai	1.8718	1.4783	1.2662	24	22	22
Ningxia	1.1507	3.2362	0.3556	26	26	27

**Table 7:** China’s agricultural environmental cost, 1996–2017.

Year	Land Erosion Cost	TN Leakage Cost	TP Leakage Cost	COD Leakage Cost	Environmental Cost
1996	13.5302	12.2438	0.8740	0.1494	26.7974
1997	13.3271	11.5935	0.7853	0.1470	25.8528
1998	12.4454	11.1079	0.7300	0.1458	24.4291
1999	12.3402	11.3967	0.8275	0.1505	24.7149
2000	12.5397	11.7116	0.8294	0.1686	25.2494
2001	12.5902	12.1051	0.8809	0.1767	25.7529
2002	12.6054	12.7126	0.9319	0.1863	26.4362
2003	10.9504	10.8697	0.8639	0.1706	22.8547
2004	11.6152	11.2822	0.9185	0.1854	24.0013
2005	11.9168	11.5364	0.9620	0.1930	24.6082
2006	12.2955	12.2594	0.9993	0.2088	25.7630
2007	13.1819	13.2230	1.1253	0.2175	27.7478
2008	13.9983	13.8679	1.2106	0.2367	29.3136
2009	15.4762	14.8507	1.2919	0.2615	31.8802
2010	16.2403	15.9221	1.4124	0.2788	33.8536
2011	18.2060	18.4126	1.6219	0.3466	38.5871
2012	18.6836	18.9991	1.7009	0.3660	39.7496
2013	20.6695	20.6412	1.9389	0.4243	43.6739
2014	21.1422	21.2275	2.0167	0.4317	44.8182
2015	20.6899	20.6089	1.9886	0.4294	43.7168
2016	20.6828	20.0064	1.9182	0.4418	43.0492
2017	19.5021	19.2296	1.8377	0.3992	40.9686
Mean	15.2104	14.8095	1.2575	0.2598	31.5372

Unit: billion yuan.

**Table 8:** Rate of agricultural green GDP to traditional GDP for 27 provinces in China.

Province	Environmental Cost/Traditional GDP	Province	Environmental Cost/Traditional GDP
Province	Green GDP/Traditional GDP	Province	Green GDP/Traditional GDP
Tibet	0.982	Inner Mongolia	0.931
Sichuan	0.970	Heilongjiang	0.928
Ningxia	0.965	Henan	0.927
Guangdong	0.962	Gansu	0.926
Anhui	0.960	Guangxi	0.925
Yunnan	0.953	Liaoning	0.921
Guizhou	0.951	Shanxi	0.920
Zhejiang	0.949	Shaanxi	0.914
Xinjiang	0.947	Fujian	0.914
Jiangxi	0.940	Hunan	0.911
Jiangsu	0.940	Hainan	0.903
Qinghai	0.940	Shandong	0.893
Jilin	0.940	Hebei	0.858

## Data Availability

The data that support the findings of this study are available from the corresponding author upon reasonable request.
